# Cirsiliol targets tyrosine kinase 2 to inhibit esophageal squamous cell carcinoma growth in vitro and in vivo

**DOI:** 10.1186/s13046-021-01903-z

**Published:** 2021-03-17

**Authors:** Xuechao Jia, Chuntian Huang, Yamei Hu, Qiong Wu, Fangfang Liu, Wenna Nie, Hanyong Chen, Xiang Li, Zigang Dong, Kangdong Liu

**Affiliations:** 1 Department of Pathophysiology, The School of Basic Medical Sciences, AMS, Zhengzhou University, 100 Kexue Avenue, Zhengzhou, 450001 Henan China; 2 China-US (Henan) Hormel Cancer Institute, Zhengzhou, 450008 Henan China; 3 The Hormel Institute, University of Minnesota, Austin, MN 55912 USA; 4 Cancer Chemoprevention International Collaboration Laboratory, Zhengzhou, Henan China; 5 Provincial Cooperative Innovation Center for Cancer Chemoprevention, Zhengzhou University, Zhengzhou, Henan China; 6 State Key Laboratory of Esophageal Cancer Prevention and Treatment, Zhengzhou, Henan China

**Keywords:** Cirsiliol, TYK2, Esophageal squamous cell carcinoma, Surface plasmon resonance, Patient-derived xenograft

## Abstract

**Background:**

Esophageal squamous cell carcinoma (ESCC) is an aggressive and lethal cancer with a low 5 year survival rate. Identification of new therapeutic targets and its inhibitors remain essential for ESCC prevention and treatment.

**Methods:**

TYK2 protein levels were checked by immunohistochemistry. The function of TYK2 in cell proliferation was investigated by MTT [(4,5-dimethylthiazol-2-yl)-2,5-diphenyltetrazolium bromide] and anchorage-independent cell growth. Computer docking, pull-down assay, surface plasmon resonance, and kinase assay were used to confirm the binding and inhibition of TYK2 by cirsiliol. Cell proliferation, western blot and patient-derived xenograft tumor model were used to determine the inhibitory effects and mechanism of cirsiliol in ESCC.

**Results:**

TYK2 was overexpressed and served as an oncogene in ESCC. Cirsiliol could bind with TYK2 and inhibit its activity, thereby decreasing dimer formation and nucleus localization of signal transducer and activator of transcription 3 (STAT3). Cirsiliol could inhibit ESCC growth in vitro and in vivo.

**Conclusions:**

TYK2 is a potential target in ESCC, and cirsiliol could inhibit ESCC by suppression of TYK2.

**Supplementary Information:**

The online version contains supplementary material available at 10.1186/s13046-021-01903-z.

## Background

Esophageal cancer is a malignant tumor worldwide, with the 7th and 6th highest incidence and mortality rates respectively [1]. Esophageal squamous cell carcinoma (ESCC) and esophageal adenocarcinoma (EAC) are two major histological types of esophageal cancer. ESCC occurred equally in the middle and lower esophagus while EAC occurred at approximately three-quarters of the distal esophagus respectively [2]. ESCC comprised over 90% of all esophageal cancer cases. Although chemotherapy, surgery, and radiotherapy are considered the most effective clinical treatments, the five-year survival rate of esophageal cancer is still less than 20% [3, 4]. Thus, the identification of effective molecular targets and their inhibitors are highly interested [5–8].

Tyrosine kinase 2 (TYK2) is a Janus kinase. It is extensively expressed in mammals. TYK2 is activated after stimulating by various cytokines [9]. After binding to its ligand, TYK2 is phosphorylated and activated at Tyr1054 or/and Tyr1055 [10]. After TYK2 activation, transcription factors such as signal transducer and activator of transcription 1 (STAT1) and STAT3, are dimerized and activated, thereby promoting the transcription of related genes and causing abnormal tumor cell proliferation and differentiation [11, 12].

Nature compounds which were found in vegetables, fruits, as well as medicinal plants which were considered as potential inhibitor resources for cancer [13, 14]. Evidence has shown that many flavonoids which are found in plants exhibit anti-cancer effects through anti-proliferation, carcinogen inactivation, induction of apoptosis, and cell cycle arrest, etc. [15, 16]. Cirsiliol, a flavonoid found in many plants such as Artemisia, Salvia, and *Leonotis nepetifolia*, has anti-inflammatory, antioxidant, antibacterial, sedative, and hypnotic effects [17–20]. It has recently been shown to exert cancer inhibition effects against non-small cell lung cancer and other cancers [21, 22]. However, the potential anti-cancer activities and the underlying mechanisms of cirsiliol in ESCC have not been fully elucidated.

In this study, we found that TYK2 served as an oncogene in ESCC and its protein level was negatively associated with ESCC patients’ overall survival rates. Cirsiliol suppressed ESCC growth through targeting TYK2/STAT3 signaling pathways.

## Materials and methods

### Materials

ESCC tissue array (Cat#HEsoS180Su05) were purchased from OUTDO Biotech (Shanghai, China). Active TYK2 (Cat#T21-11G) and inactive STAT3 protein (Cat#S54-54BH) were bought from Signal Chem company (Richmond, BC, Canada). Cirsiloil (Cat#FD66719) was purchased from Carbosynth (Suzhou, China). Antibodies to detect p-STAT3 (Cat#9145), STAT3 (Cat#9139), Bcl-2 (Cat#15071), GAPDH (Cat#2118), myeloid cell leukemia-1 (Mcl-1, Cat#94296), PARP (Cat#9542) and β-actin (Cat#3700) were all purchased from CST antibody company (Beverly, MA, USA). TYK2 (Cat#ab223733) and Ki67 (Cat#ab16667) were purchased from abcam (Cambridge, MA, USA). NativePAGE™ Bis-Tris Gel (Cat#BN1002BOX) and NativePAGE™ Sample Buffer (Cat#BN2003) were purchased from ThermoFisher (Waltham, MA, USA). Cytoplasmic and Nuclear Protein Extraction Kit was bought from Beyotime (Cat#P0028, Shanghai, China). BCA kit was purchased from Solarbio (Cat#PC0020, Beijing, China).

### Cell culture and proliferation assay

Human ESCC cell lines KYSE30, KYSE70, KYSE140, KYSE410, KYSE450 and KYSE510 were bought from the Type Culture Collection of Chinese Academy of Sciences (Shanghai, China) and the cell lines were mycoplasma-free and authenticated by STR analysis. The cancer cells were cultured in RPMI-1640 media with 10% fetal bovine serum. The immortalized normal human esophageal epithelial cell-SHEE was donated by Professor Enmin Li. Cells were seeded at 1.5–6 × 10^3^ cells/well in 96-well plates, incubated no less than 12 h, and then added with a series concentrations of cirsiliol or vehicle. After incubation for an additional 24, 48, or 72 h, the proliferation of cell was detected using MTT (0.5 mg/mL) reagent.

### Anchorage-independent cell growth and cell cycle analysis

After preparing the 0.5% base layer agar with vehicle, 5, 10 or 20 μM cirsiliol, KYSE140 and KYSE450 cells (8 × 10^3^ cells/well) were seeded in a 0.3% top layer agar with vehicle, 5, 10 or 20 μM cirsiliol. The cells were cultured in an incubator for an additional 2 weeks. The colonies were photographed by a microscope and counted by Image-Pro Plus v6.0. To analyzing cell cycle, ESCC cells were cultured in 60 mm dishes and treated with 0, 5, 10 or 20 μM cirsiliol for 48 h. After fixation in 70% pre-cooled ethanol for 24 h and incubation with propidium iodide, the cells were detected using flow cytometer (BD Biosciences, San Jose, CA).

### Cell migration

KYSE140 and KYSE450 cells (2 × 10^5^ cells) resuspended in 200 μl RPMI-1640 media were seeded in the upper chamber of transwell plate insert (Cat#3422, Corning, USA). The down chamber part was added with 600 μl 10% FBS RPMI-1640 media. After culture for 24 h, the inserts were washed twice with PBS. After fixing with methanol, cells were stained with 500 μl 0.1% crystal violet, which, after imaging with an inverted microscope, were eluted with 33% acetic acid. The migration cells were quantified by measuring the absorbance at 570 nm.

### Plasmid mutation and TYK2 overexpression in 293 T cell

The TYK2 pcDNA3.1–3 × Flag-C plasmid was purchased from Youbao Biotechnology Company (Changsha, China). The TYK2 mutation information showed as follow: 1A (Val 981 to Ala); 1’A (Pro 982 to Ala); 2A (double amino mutation to Ala). The primers were showed in Table 1. The mutation was performed according the fast mutagenesis system kit (Cat#FM111, Trans, China). After transfected the wild type and mutant plasmid into 293 T cell for 48 h, the cells were harvested and prepared lysates for the pull down assay.

**Table 1:** The site mutant primers of TYK2

Name	Forward primer (5′ to 3′)	Revers primer (5′ to 3′)
1A	CTGGTCATGGAGTACGCGCCCCTGGGCA	GCGTACTCCATGACCAGCTGCAGCGACT
1’A	GGTCATGGAGTACGTGGCCCTGGGCAGC	CCACGTACTCCATGACCAGCTGCAGCGA
2A	GGTCATGGAGTACGCGGCCCTGGGCAGC	CCGCGTACTCCATGACCAGCTGCAGCGA

1A (Val 981 to Ala); 1’A (Pro 982 to Ala); 2A (double mutant to Ala)

### Lentiviral infection and transfection

The viral and packaging vectors (pMD2.G, pLKO.1-mock, psPAX2, and shTYK2) were co- transfected with Simple-Fect Transfection Reagent (Signaling Dawn Biotech, Wuhan, China) into 293 T cells. The shRNA sequences of TYK2 were shown as bellow: shTYK2#2-F: 5’CCGGGAGATCCACCACTTTAAGAATCTCGAGATTCTTAAAGTGGTGGATCTCTTTTTG3’, R: 5’AATTCAAAAAGAGATCCACCACTTTAAGAATCTCGAGATTCTTAAAGTGGTGGATCTC3’; shTYK2#3-F: 5’CCGGCGAGCACATCATCAAGTACAACTCGAGTTGTACTTGATGATGTGCTCGTTTTTG3’, R: 5’AATTCAAAAACGAGCACATCATCAAGTACAACTCGAGTTGTACTTGATG ATGTGCTCG3’. Viral particles were collected 48 h after transfection using a 0.45 μm filter. After infecting with 8 μg/mL polybrene virus particles media (Millipore, Billerica, MA) for another 24 h, the cells were selected with 4 μg/mL puromycin and then used for subsequent experiments.

### Computational docking model

We conducted the *in-silico* docking assay through using the Schrödinger Suite 2015 to determine whether cirsiliol could bind to TYK2. The crystal structure of TYK2 was downloaded from the protein databank and prepared following the standard methods of Protein Preparation Wizard (Schrödinger Suite 2015). After removing all water molecules, the hydrogen atoms were adjusted at a pH of 7. Cirsiliol was used for docking following the default parameters of LigPrep program. Then, cirsiliol was docked to TYK2 using the extra precision mode of Glide.

### Pull-down assay

The Cyanogen bromide (CNBr)-cirsiliol-coupling Sepharose beads were prepared according to the CNBr-activated Sepharose 4B (Cat#71–7086-00AF, GE Healthcare, MA, USA) specification [23]. Cell proteins (500 μg) were rotated with 50 μl cirsiliol-Sepharose 4B beads or vehicle in the reaction buffer. After gentle rocking for 15 h at 4 °C, the conjugated beads and vehicle were washed 4 times in the washing buffer followed by the addition of 30 μl 1× loading buffer at 95 °C for 10 min. The binding was assessed using western blotting.

### Surface plasmon resonance (SPR)

SPR was conducted with a Biacore T200 instrument (GE Healthcare, MA, USA). TYK2 (20 μg/mL) was covalently immobilized on a CM5 chip (Cat#BR-1005-30, GE Healthcare) across ligand flow channels. Then, the chip was equilibrated with PBS. A concentration series of cirsiliol were added into the flow system to test the binding affinity between cirsiliol and TYK2. Cirsiliol was dissolved in PBS with 0.1% DMSO, 30 μl/min flow rate, 120 s contact time, and 300 s dissociation time were set. The software of T200 evaluation state model was utilized to analyze the binding affinity data and calculated the compound’s KD value. Representative curves were re-plotted in GraphPad Prism.

### In vitro kinase assay

Inactive STAT3 proteins (1 μg) were incubated with 200 ng active TYK2 (442-end) for in vitro kinase assays. The reactions were conducted in kinase buffer II (Cat#K02–09, SignalChem, Canada) containing 200 μM adenosine triphosphate (ATP). Different concentrations of cirsiliol were added (final concentration 0, 5, 10, 20 μM), and the samples were maintained at 30 °C for 30 min. The kinase reactions were terminated using 5 μl 6× loading buffer and heating for 10 min at 95 °C. The proteins were detected using western blotting.

### Cell immunofluorescence assay

First, we placed sterile glass coverslips into 24 well plates. Next, 2 × 10^4^ KYSE140 and KYSE450 cells were seeded into the wells. After attachment to the surface of the coverslips, cells were cultured with 0, 5, 10 or 20 μM cirsiliol for 24 h. After washing in PBS, the cells were fixed using 4% paraformaldehyde for 30 min. Following another 3 times washes in PBS, special primary antibody were added into the cells at 4 °C for 15 h and then incubated in the second fluorescent antibodies with diisopropylamine (DIPA) at room temperature for 2 h. After washed by PBS, the coverslips were transferred to glass slides with an anti-fluorescence quenching agent. The results were analyzed using Image-Pro Plus v6.0.

### Native gel electrophoresis

The Native gel electrophoresis assay was conducted based on the Gel System protocol of the NativePAGE™ Novex® Bis-Tris. Cells were incubated for 30 min on ice in 1× sample buffer (50 mM Bis-Tris, 0.1% n-dodecyl-β-D-maltoside, 6 N HCl, 50 mM NaCl, 0.001% Ponceau S, 10% Glycerol, pH 7.2). After 30 min 20,000 *g* centrifugation, the supernatant was collected, and the concentration was measured using BCA kit. NativePAGE™ gel (4–16%) electrophoresis was performed at 150 V constant voltages. After running for 30 min the Cathode Buffer-Dark Blue was changed with Light Blue and electrophoresis was resumed for another 80 min. The gel was then transferred in 1 × NuPAGE® Transfer Buffer at 100 mA constant for 1 h and the membrane was fixed with 20 mL of 8% acetic acid for 15 min and immunodetection was directly performed.

### Nuclear and cytoplasmic protein extraction

We utilized the Nuclear and Cytoplasmic Protein Extraction kit to analyze the STAT3 nuclear translocation. After harvesting ESCC cells treated with cirsiliol for 24 h, we extracted nuclear proteins according to the instructions of manufacturer. The STAT3 protein levels in nuclear and cytoplasm extracts were subsequently quantified using the BCA kit and detected via immunodetection.

### Western blotting

Cells were suspended in cell lysis buffer (50 mM Tris, 150 mM NaCl, 1% NP-40, and 1 mM phenylmethylsulphonyl fluoride, pH 8.0) and incubated on ice for 30 min. After centrifugation (12,000 g, 10 min), the supernatant were collected as whole cell extracts. The concentration of the extracts was measured using a BCA kit. Via gel electrophoresis and transfer, the extracts were transferred to polyvinylidene fluoride membranes. After blocking with 5% non-fat milk in 1 × PBS, the membranes were incubated with TYK2, p-STAT3, STAT3, c-myc, Bcl-2, Mcl-1 or GAPDH antibodies. These proteins on the membrane were detected using a chemiluminescence reagent.

### CDX mouse model

KYSE450 cells were utilized to prepare the CDX mouse model. First, the cells were infected with the mock, shTYK2#2, and shTYK2#3 packaged virus. After selection by puromycin, cells were expanded in culture. After acquiring sufficient numbers of cells, 1 × 10^7^ KYSE450 cells were harvested and seeded in the right flank of each mouse. After 3 weeks, the volume of the tumors was measured every 2 days. The tumor weight was measured when the tumor volume reached 1 cm^3^.

### PDX mouse model

Six weeks old female severe combined immunodeficient (SCID) mice were bought from Vital River Labs (Beijing, China) and kept in a 12/12 h light/dark cycle condition with free access to food and water. The PDX tumor tissue was cut into fragments of about 1–2 mm and implanted into the mouse’s right flank. When the average tumor volume reached about 100 mm^3^, the mice were randomly divided into 3 groups (*n* = 9 per group): vehicle group; middle dose group (10 mg/kg cirsiliol); high dose group (50 mg/kg cirsiliol). Cirsiliol or vehicle (5% DMSO and 20% PEG400 in PBS) was administered by gavage once daily. The tumor volume and body weight of each mice were checked twice every week. The PDX mice were monitored until the tumor volume reached 1 cm^3^, and then the tumors were extracted after the mice were euthanized.

### Immunohistochemistry (IHC) analysis

Tumor tissues embedded in paraffin were used for IHC staining. After deparaffinization, antigen unmasking, and blocking by 5% goat serum for 40 min at room temperature, the slices were incubated with antibodies against Ki-67, p-STAT3 (Tyr705), and TYK2 at 4 °C for 15 h, and then incubated with the secondary antibody followed by DAB (3, 3′-diaminobenzidine) staining. After counter-staining and dehydration, the slices were mounted on glass coverslips with neutral resin. The slices were photographed (100 × magnification) and analyzed using the Aperio ImageScope software program.

### Statistical analysis

In this study, GraphPad Prism8.0 was used to conduct all statistical analysis and quantitative results were showed as mean ± SD. The unpaired Student’s *t*-test or one-way analysis of variance (ANOVA) was used to compare significant differences. *p < 0.05, **p < 0.01, and ***p < 0.001 were used to show significance for each experiment.

## Results

### TYK2 is highly expressed in ESCC and negatively associated with patient survival

**Fig. 1:**
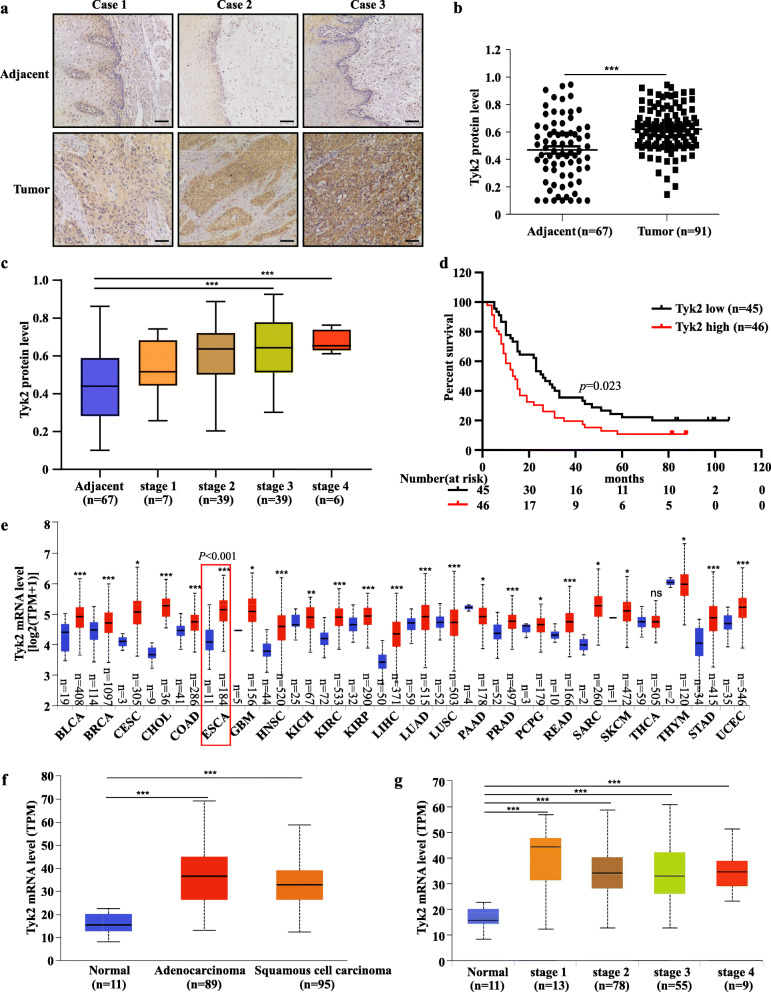
TYK2 expression is higher in ESCC and is negatively associated with patient survival. **a** Representative IHC staining images of ESCC tissue array using specific antibody for TYK2 in adjacent tissues and paired cancer tissues. Scale bar: 50 μm. **b** Analysis of TYK2 protein levels in ESCC tissue array according to staining results. TYK2 expression was valued as positivity. **c** Protein levels of TYK2 in ESCC tissue array based on clinical stages. **d** Survival rates of cancer patients with high or low protein levels of TYK2 in ESCC tumor microarray. Kaplan-Meier method was used to generate the survival curve. *P* < 0.05 was considered to be statistically significant. **e** The mRNA levels of TYK2 in different cancers based on the TCGA database. **f** The mRNA levels of TYK2 in ESCA (TCGA database) based on tumor histology. **g** The mRNA levels of TYK2 in ESCA (TCGA database) based on clinical stages. Due to some of the clinical stage information missing, the cases number in (**g**) is not the same with (**f**). Statistical analysis was performed using Student’s unpaired *t*-test in (**b**, **c**, **e**, and **f**); ANOVA in (**g**). Asterisks (*p < 0.05, **p < 0.01, ***p < 0.001) indicate a significant change. TPM, transcription per million

The protein levels of TYK2 were evaluated by IHC staining in ESCC tissue array (Fig. 1a). The results showed that TYK2 protein levels were higher in tumors than in adjacent tissue (Fig. 1b). TYK2 protein levels were significantly increased in stage 3 and stage 4 compared with adjacent tissues (Fig. 1c). A lower survival rate was also observed in TYK2 protein level highly ESCC patients (Fig. 1d). Then we utilized UALCAN to assess the gene information in the TCGA database [24]. In the UALCAN data base, bioinformatics analysis showed that the TYK2 mRNA level was significantly up-regulated both in EAC and ESCC (Fig. 1f). Furthermore, TYK2 mRNA was also up-regulated in other 22 kinds of cancers (Fig. 1e). Similarly, TYK2 mRNA level in UALCAN data base was significantly up-regulated in all ESCC tumors stages (Fig. 1g). The correlation between TYK2 protein level and ESCC tissue array clinic pathologic characteristics are shown in Table 2.

**Table 2:** Cohort characteristics of esophageal cancer patients

Clinicopathological characteristics	TYK2 protein expression level		
	Low (***n*** = 45)	High (***n*** = 46)	***P***
Gender			
Male	29 (64.4%)	39 (84.8%)	0.113
Female	16 (35.6%)	7 (15.2%)	
Age			
≤ 60	14 (31.1%)	17 (37.0%)	0.761
> 60	31 (68.9%)	29 (63.0%)	
Tumor size			
≤ 5 cm	31 (68.9%)	25 (54.3%)	0.528
> 5 cm	14 (31.1%)	21 (45.7%)	
Histological grade			
Well/moderately	30 (66.7%)	37 (80.4%)	0.355
Poorly	15 (33.3%)	9 (19.6%)	
T classification			
T1	4 (8.9%)	2 (4.3%)	0.543
T2	7 (15.6%)	5 (10.9%)	
T3	32 (71.1%)	38 (82.6%)	
T4	2 (4.4%)	1 (2.2%)	
N classification			
N0	22 (48.9%)	19 (41.3%)	0.448
N1	16 (35.6%)	14 (30.4%)	
N2	6 (13.3%)	10 (21.8%)	
N3	1 (2.2%)	3 (6.5%)	
Clinical stage			
1	5 (11.1%)	2 (4.4%)	0.033
2	20 (44.5%)	19 (41.3%)	
3	18 (40.0%)	21 (45.6%)	
4	2 (4.4%)	4 (8.7%)	

### Knocking down of TYK2 suppressed the growth of ESCC

**Fig. 2:**
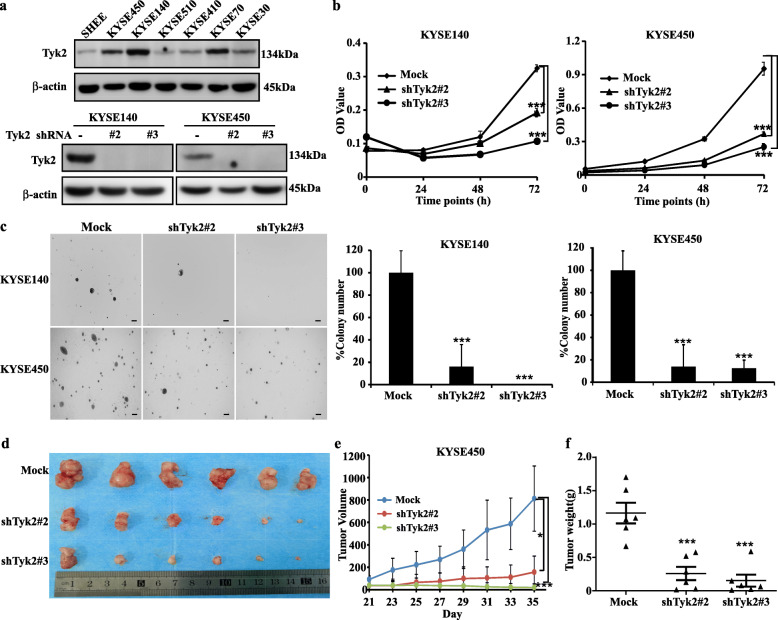
TYK2 knockdown suppresses ESCC growth. **a** Upper panel: TYK2 protein level in the normal esophagus and ESCC cell lines; lower panel: TYK2 knockdown results in KYSE140 and KYSE450 cells. **b** Left panel: KYSE140 cell viability; right panel: KYSE450 cell viability. **c** Left panel: the representative colony images of ESCC cell lines. Scale bar: 200 μm. Middle panel: Statistical analysis of the clone ratio in KYSE140 cells. Right panel: Statistical analysis of the clone ratio in KYSE450 cells. **d** The tumor pictures of the cell-derived tumor xenograft (CDX) mouse model. **e** The tumor growth status after transplanted TYK2 knockdown cells in the CDX mouse model. **f** The analysis of tumor weight of the CDX mouse. Student’s unpaired *t*-test was used in (**b**, **c**, **e**, and **f**). Asterisks (*p < 0.05, ***p < 0.001) indicate a significant change

To evaluate the role of TYK2 in ESCC, we checked the protein level of TYK2 in immortalized esophagus cell SHEE and ESCC cell lines. The results showed that TYK2 protein levels in ESCC Cell lines were higher than immortalized esophagus cell (Fig. 2a, up panel). Then highly expressed TYK2 KYSE140 and KYSE450 cell lines were selected for further knockdown assays. Results showed that shTYK2#2 and shTYK2#3 decreased the protein levels of TYK2 notably in both cell lines (Fig. 2a, down panel). After knocking down of TYK2, the growth of KYSE140 and KYSE450 were suppressed (Fig. 2b). Similarly, the anchor independent cell growth was also attenuated in the shTYK2#2 and shTYK2#3 groups (Fig. 2c). Furthermore, the tumor growth of Cell-derived xenograft (CDX) mice model was reduced after TYK2 knockdown (Fig. 2d & e and Supplementary 1a) and the average of tumor weight in the TYK2 knockdown group was lower than the control group in the CDX mice model (Fig. 2f). In addition, after TYK2 knockdown, the cell cycle was arrested at the G2/M phase in both KYSE140 and KYSE450 cell lines (Supplementary 1b). Similarly, cell migration was also decreased following TYK2 knockdown in KYSE140 and KYSE450 (Supplementary 1c).

**Fig. 3:**
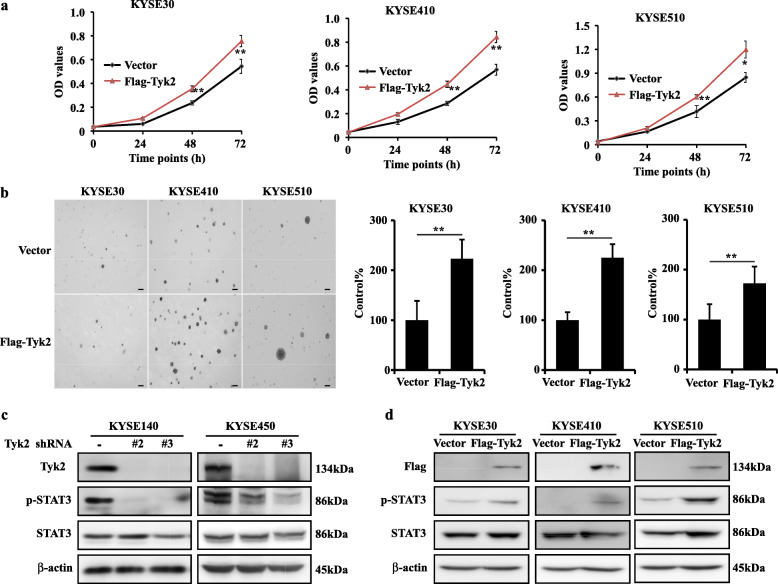
Overexpressed TYK2 increased ESCC growth. **a** Cell viability of KYSE30, KYSE410 and KYSE510 after transfection with TYK2. **b** The representative colony images and Statistical analysis of ESCC cell lines. Scale bar: 200 μm. **c** The change of downstream signaling after TYK2 knockdown in KYSE140 and KYSE450 cells. **d** The change of downstream signaling after TYK2 overexpressed in KYSE30, KYSE410 and KYSE510 cells. Student’s unpaired *t*-test was used in (**a** and **b**). Asterisks (*p < 0.05, **p < 0.01) indicate a significant change

Then, we overexpressed TYK2 in KYSE30, KYSE410 and KYSE510 cell lines. After transfected with pcDNA3.1-TYK2–3 × Flag or vehicle plasmid for 36 h, cells were seeded for cell proliferation and anchorage-independent cell growth assay. Results showed that the cell proliferation and cell colony formation were increased (Fig. 3a and b). For the related mechanism study, we found that the STAT3 phosphorylation was inhibited after TYK2 knockdown in KYSE140 and KYSE450 cell lines (Fig. 3c). Similarly, western blotting results also showed overexpressing TYK2 could stimulate the phosphorylation of STAT3 in KYSE30, KYSE410 and KYSE510 cells (Fig. 3d). In summary, these data indicated that TYK2 played an oncogenic role in ESCC growth.

### TYK2 is a target of cirsiliol

**Fig. 4:**
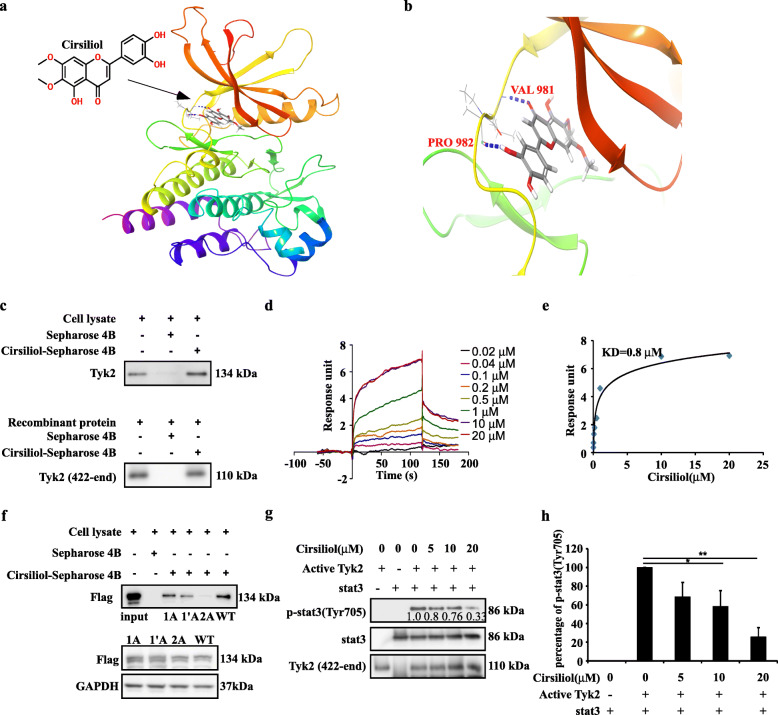
Cirsiliol binds with TYK2 and inhibits the kinase activity. **a** Computational docking model between cirsiliol and TYK2. **b** The detailed binding site of cirsiliol on TYK2. **c** Upper panel: the binding ability of cirsiliol on endogenic TYK2 in vitro*,* obtained via pull down assay. Down panel: the binding ability of cirsiliol to recombinant TYK2 protein. **d** The change of affinity response intensity with the passage of time. -60 to 0 s was set as the time before inject cirsiliol solution; 0–120 s was set as contact time between cirsiliol and TYK2; 120–180 s was set as dissociation time. **e** The variation of response intensity with the increase of cirsiliol concentration. **f** Upper panel: cirsiliol binding ability with mutant TYK2. Down panel: the protein level of TYK2 in 293 T cell line. 1A (V981A), 1’A (P982A), 2A (double mutant), WT: wild type. **g** Kinase assay performed with cirsiliol and TYK2. **h** p-STAT3 inhibition analyzed by ImageJ in three independent assays (*n* = 3, *p < 0.05, **p < 0.01). Student’s unpaired *t*-test in (**h**). KD, dissociation constant

Since TYK2 had a positive effect on ESCC proliferation, we attempted to identify an inhibitor of TYK2; hence we used an *in-silico* docking assay to screen compounds from natural compound library. Our results showed that cirsiliol could bind to TYK2. The chemical structure of cirsiliol and cirsiliol-TYK2 docking model are shown in Fig. 4a. The results predicted that cirsiliol achieves binding at PRO982 and VAL981 in the ATP binding pocket (Fig. 4b). Furthermore, pull-down assay showed that Sepharose 4B-coupled-cirsiliol could bind directly to endogenous and recombinant TYK2 protein ex vitro (Fig. 4c). Similarly, SPR assay also showed that the binding affinity between cirsiliol and TYK2 protein increased over time in a concentration dependent manner (Fig. 4d). The determined equilibrium dissociation constant (KD) of cirsiliol was approximately 0.8 μM (Fig. 4e). We then evaluated whether PRO982 and VAL981 docking sites were important for the binding ability of cirsiliol. After single and double mutation, the amino sites, the cell lysate were harvested for pull down assay, which showed that the binding ability of cirsiliol was decreased after mutating the docking site to Alanine (Fig. 4f). In addition, kinase activity assay was utilized to detect the inhibitory effects of cirsiliol on TYK2 activity. Results showed that cirsiliol significantly suppressed the activity of TYK2 at concentrations of 10 μM and 20 μM (Fig. 4g & h).

### Cirsiliol suppresses ESCC growth

**Fig. 5:**
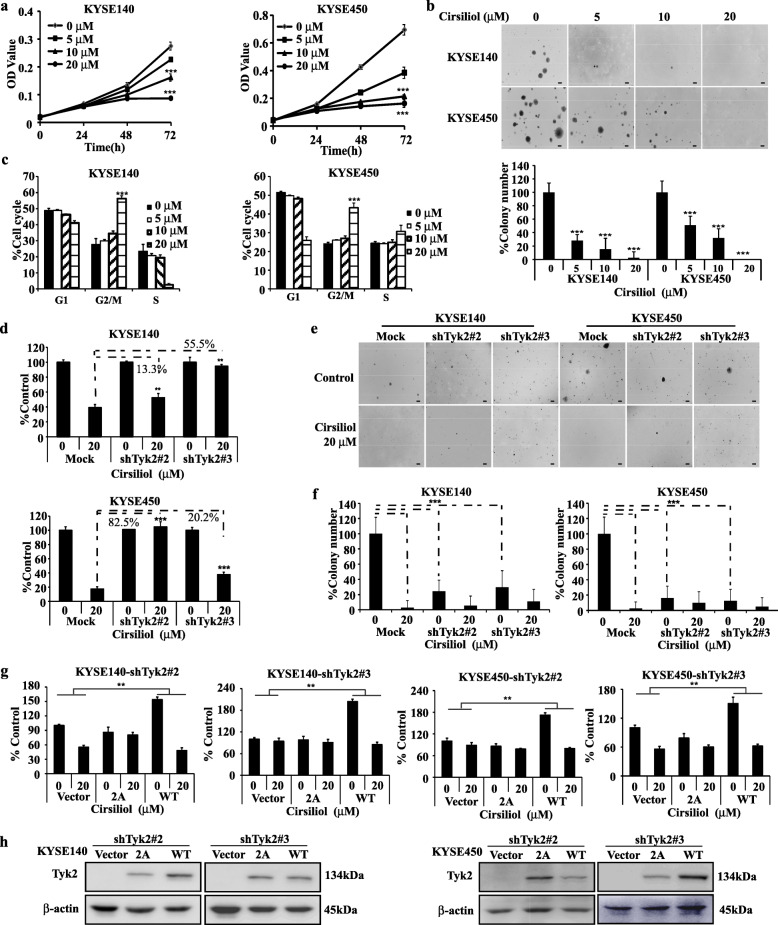
Cirsiliol inhibits ESCC cell proliferation and the inhibiting effect is attenuated when TYK2 knockdown. **a** MTT assay showed that cirsiliol suppresses KYSE140 (left panel) and KYSE450 (right panel) cell viability. **b** Colony forming assay. Upper panel: the representative clone pictures of KYSE450 and KYSE140, scale bar: 200 µm; lower panel: Statistical analysis of the colony ratios of both cell lines after cirsiliol treatment. **c** Cell cycle arrest at the G2/M phase after cirsiliol treatment. Left panel: KYSE140 cells; right panel: KYSE450 cells. **d** The proliferation inhibiting ability of cirsiliol change after TYK2 knockdown (three independent groups were set up in each cell line and the data was analyzed between vehicle and treatment in each group separately). Upper panel: KYSE140 cells; lower panel: KYSE450 cells. Scale bar: 200 µm. **e** Representative colony pictures after TYK2 silencing with or without cirsiliol treatment. **f** Analysis of the colony number. Cell colonies were analyzed using Image-Pro Plus v6.0. **g** Cell viability after rescue TYK2 protein level in KYSE140 and KYSE450 TYK2 knockdown cell lines. **h** The change of TYK2 protein level and phosphorylation STAT3 after rescue TYK2 in KYSE140 and KYSE450. Student’s unpaired *t*-test was used in (**a**, **b**, **c**, **f**, **g**) and ANOVA in (**d**). Asterisks (**p < 0.01, ***p < 0.001) indicate a significant change

Cell proliferation and anchorage-independent cell growth assays were performed to confirm the anti-cancer effects of cirsiliol. Results revealed that cirsiliol (20 μM) inhibited KYSE140 cell proliferation by 36.2 and 68.5% at 48 and 72 h respectively, inhibited KYSE450 cell proliferation of by 48.2 and 76.4% at 48 and 72 h, respectively (Fig. 5a). Similarly, the colony counts of KYSE140 and KYSE450 were both decreased in a dose-dependent pattern after cirsiliol treatment (Fig. 5b). Next, we checked the cell cycle distribution after cirsiliol treatment. Compared to the untreated group, results showed that the cell cycle of KYSE140 and KYSE450 was arrested at the G2/M phase (56.3 and 43.4% at 20 μM, respectively; Fig. 5c). Nevertheless, cirsiliol showed less toxicity on immortalized esophagus cell SHEE at this concentration (Supplementary 1d).

### Cirsiliol inhibitory effects on ESCC partly depend on TYK2

Our data indicated that cirsiliol could effectively inhibit the proliferation and colony formation of ESCC cells. However, it remains unknown whether the inhibitory effect of cirsiliol is dependent on expression of TYK2. Thus, we treated TYK2 knockdown ESCC cells with cirsiliol. After treatment with cirsiliol for 72 h, cell viability was assessed in KYSE140 and KYSE450 cell lines in mock, shTYK2#2 and shTYK2#3 groups to evaluate their sensitivity to cirsiliol. Results indicated that the cell viability of KYSE140 with cirsiliol treatment in the mock group was 13.3, 55.5% lower than those of shTYK2#2 and shTYK2#3 groups, respectively (Fig. 5d upper panel). Similarly, compared with the mock group of KYSE450, the cell viability of shTYK2#2 and shTYK2#3 was increased by 82.5 and 20.2% after cirsiliol treatment, respectively (Fig. 5d lower panel). Moreover, the inhibitory effect of cirsiliol on clone formation was also weakened after TYK2 knockdown (Fig. 5e & f). Then, we rescued the knockdown cells with vehicle, wild type TYK2, and double mutant TYK2. After transfected for 36 h, cell proliferation assay and western blot were conducted. Results showed that after transfected with wild type TYK2, cells sensitivity to cirsiliol was increased when compared with vehicle (Fig. 5g), however, no significant changes were observed in cell proliferation ability and sensitivity in the mutant group. Western blot results showed that after rescuing TYK2 in knockdown cells, wild type and mutant type TYK2 both overexpressed in knockdown cell lines (Fig. 5h).

### Cirsiliol inhibits TYK2 downstream signaling

**Fig. 6:**
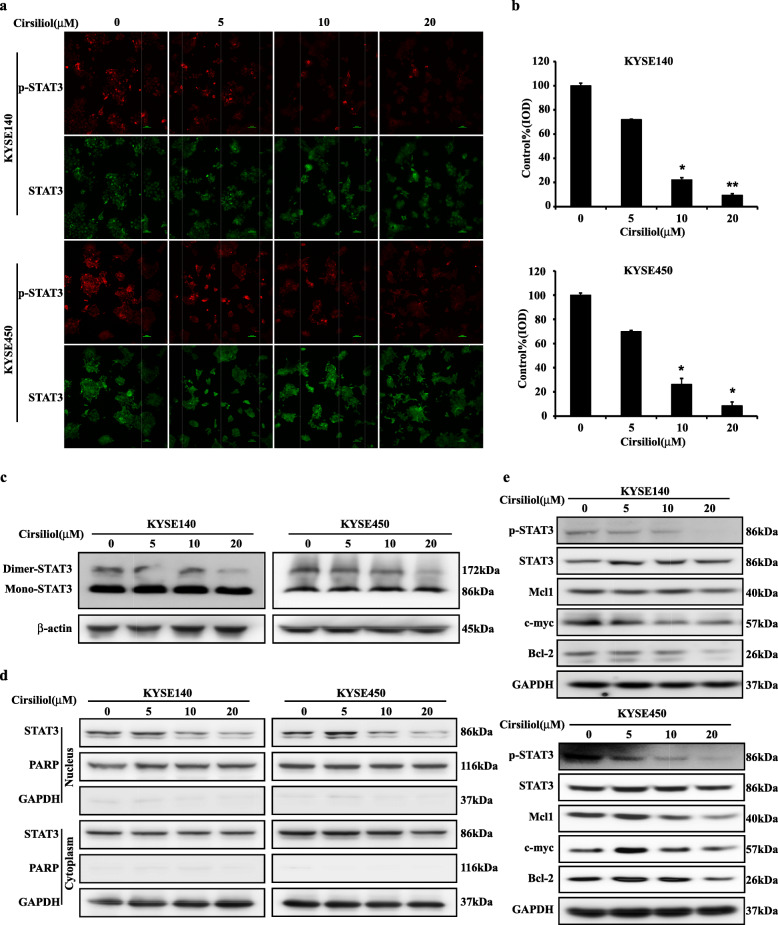
Cirsiliol inhibits ESCC through TYK2-STAT3 signaling pathways. **a** Immunofluorescence staining of KYSE140 and KYSE450: cells were treated for 24 h, and then stained for p-STAT3 (Tyr705) and STAT3 (100× magnification). **b** The analysis of p-STAT3 fluorescence intensity in KYSE140 and KYSE450 cells. Analyzed by student’s unpaired *t*-test (*p < 0.05, **p < 0.01). **c** The change of STAT3 dimer formation after treated by cirsiliol in KYSE140 and KYSE450 cells. **d** The Nucleus localization variation of STAT3 after treated with 20 μM cirsiliol in KYSE140 and KYSE450 cells. **e** The effects of cirsiliol on the TYK2-related signal pathway in KYSE140 and KYSE450 cells. IOD, Integrated Optical Density

Our previous data indicates that cirsiliol can inhibit the activity of TYK2 in vivo; hence, we investigated the downstream signaling molecule of TYK2. We performed immunofluorescence assays to detect the variation of STAT3 (Tyr705) phosphorylation and total STAT3. Results showed that STAT3 (Tyr705) phosphorylation was inhibited after cirsiliol treatment while the total STAT3 signal did not change (Fig. 6a & b). In addition, we utilized native gel electrophoresis to investigate the change in STAT3 dimerization. Results showed that STAT3 dimerization was decreased in both KYSE140 and KYSE450 cell lines after cirsiliol treatment (Fig. 6c). The cytoplasmic and nuclear proteins extracting assay was performed to evaluate the STAT3 localization change after cirsiliol treatment. Results showed that fewer STAT3 was present in the cell nucleus than the control group after cirsiliol treatment in both cell lines (Fig. 6d). Furthermore, western blotting was conducted to detect the signaling pathway associated with TYK2. The results showed that cirsiliol inhibited the phosphorylation of STAT3 in both KYSE140 and KYSE450 cells. The expression levels of STAT3-targeting gene, including Mcl-1, c-myc, and cyclin D1, were decreased in a dose-dependent pattern after cirsiliol treatment (Fig. 6e). Thus, the above data supported that cirsiliol inhibited ESCC by blocking the TYK2/STAT3 pathway.

### Cirsiliol inhibits ESCC patient-derived xenograft (PDX) growth

**Fig. 7:**
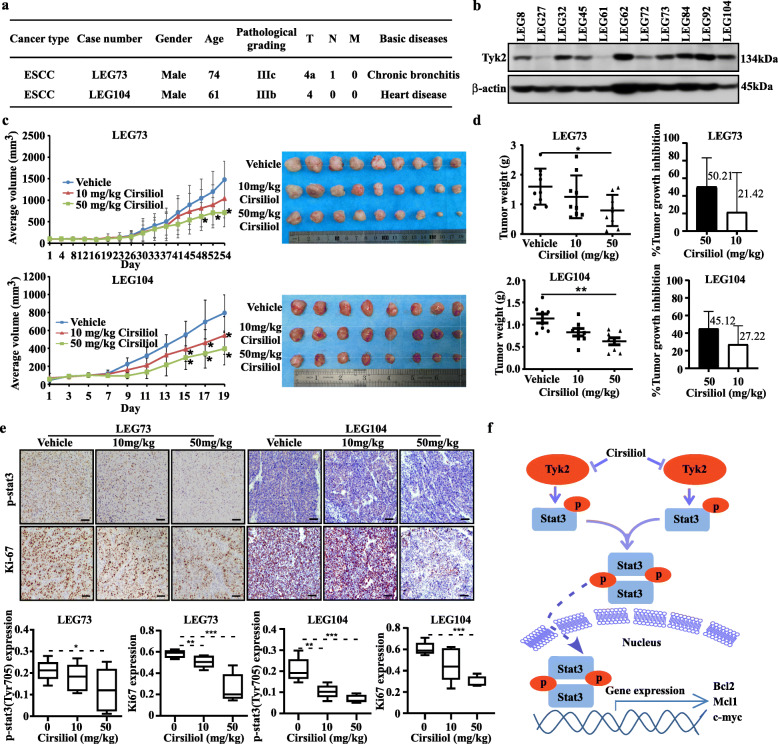
Cirsiliol inhibits ESCC patient-derived xenograft (PDX) tumor growth in vivo. **a** The information of two PDX cases. **b** The protein levels of TYK2 in different PDX cases. **c** The change of average tumor volume in different group of LEG73 and LEG104 cases after cirsiliol treatment (*p < 0.05) and tumor images of different groups after sacrifice. **d** Tumor weight analysis in different groups of LEG73 and LEG104 cases after cirsiliol treatment and the tumor growth inhibition of cirsiliol compared with the average tumor weight of the vehicle group (*p < 0.05, **p < 0.01). **e** Upper panel: Representative IHC images of LEG73 and LEG104 tumor tissue slices (100 × magnifications), tumor tissues were stained with p-STAT3 and anti-Ki67; lower panel: Statistical analysis of IHC positive staining of p-STAT3 and Ki67 in both LEG73 and LEG104 cases (*p < 0.05, **p < 0.01, ***p < 0.001). **f** Schematic diagram of the signaling pathway of esophageal cancer proliferation inhibited by cirsiliol. Data for each treatment group were compared to the control group and statistical significance was analyzed by student’s unpaired *t*-test

In order to assess the anti-tumor activity of cirsiliol in vivo, LEG73 and LEG104 cases with high level of TYK2 were selected from the ESCC PDX specimen repository to develop the PDX mouse model (Fig. 7a & b). After administration of cirsiliol via oral gavage, the tumor volumes of high cirsiliol concentration treatment group in LEG73 case were suppressed when compared with the vehicle group (Fig. 7c). Similarly, the tumor volumes of both cirsiliol treatment groups in LEG104 case were also reduced (Fig. 7c). After sacrificing the mice and harvesting tumors, the tumor pictures in both cases were shown in Fig. 7c. The average tumor weights of high cirsiliol concentration group in both cases were obviously lower than vehicle group (Fig. 7d). After analyzing the tumor weights in each group, the average tumor growth inhibition rate of the high concentration treatment group in the two cases were 50.21 and 45.12% compared with the vehicle group, respectively (Fig. 7d). Furthermore, the tumors were stained with Ki-67 and p-STAT3 specific antibody for mechanism study after tissue slice preparation. The representative IHC staining images are shown in Fig. 6e. IHC analysis revealed that cirsiliol inhibited the phosphorylation of STAT3 and reduced Ki-67 protein level significantly after cirsiliol treatment (50 mg/kg) in LEG73 and LEG104 cases (Fig. 7e). Mice’s body weights were not significantly changed between each group after cirsiliol treatment (Supplementary 1e). The acute toxicity test results showed that cirsiliol had no effects on the mice body weight and white blood cell when comparing with vehicle group at the concentration of 50 mg/kg (Supplementary 1f).

## Discussion

Esophageal squamous cell carcinoma is an aggressive cancer worldwide. Due to lack of effective therapeutic targets and related drugs, the five-year survival rate of ESCC is still less than 20%. Thus, finding effective therapeutic targets and inhibitors for ESCC is highly interested.

Previous studies showed that TYK2 is overexpressed in several cancer cells and be supposed to a potential therapeutic target [25, 26]. In Malignant peripheral nerve sheath tumors (MPNST), TYK2 induces cell proliferation and promotes MPNST progression through inhibiting cell death [27]. In prostate cancer, TYK2 influences the invasiveness of prostate cancer cells [28], While, in osteosarcoma cell lines, TYK2 is essential for cancer cell survival [29]. Furthermore, TYK2 inhibition could also block the invasiveness of breast cancer cells [30]. Herein, we investigated the role of TYK2 in ESCC and found that the mRNA and protein levels of TYK2 were expressed highly in the most tumor tissues (Fig. 1b-e). The tissue array results showed that TYK2 protein levels were positively correlated with the tumor clinical stage while it was negatively correlated with patient survival rate in ESCC (Fig. 1c-d). However, due to the limited clinical samples, we could not validate the relationship between TYK2 and patient survival rate using the TCGA data base. Also, since TYK2 protein level differs in various adjacent tissues, potential side-effects for later target therapy may be a challenge. Despite this limitation, our findings illustrate the clinical significance of our research. Combined with TYK2 knockdown and overexpression assays in ESCC cell lines, results confirmed the positive role of TYK2 in ESCC proliferation.

Previous reports showed that cirsiliol induced radio sensitization in non-small cell lung cancer cell lines and inhibited interleukin (IL)-6-incuded STAT3 activation [22, 31], and that cirsiliol could restrain the colony formation and migration of melanoma cells [32]. However, the underlying mechanisms and related targets have not been elucidated. In the present study, we utilized computational docking model to exhibit that cirsiliol could bind to TYK2 (Fig. 4). In addition, we also confirmed this by using pulldown assay and SPR assay (Fig. 4). Furthermore, utilizing kinase assay, we verified that cirsiliol inhibited the kinase activity of TYK2 in vitro rather than detecting the signal only in cell lines. These data show that cirsiliol is an inhibitor of TYK2.

PDX models have high stability and resemblance to human tumors. Thus, they are the preferred translational tools in preclinical studies and widely applied in drug discovery [33]. To evaluate the inhibitory effects of cirsiliol on ESCC, two cases of ESCC PDX models which expressed TYK2 at high levels were selected for our in vivo study. Results showed that cirsiliol decreased both tumor volume and tumor weight obviously (Fig. 7c-d). IHC staining of Ki67 and p-STAT3 showed that cirsiliol significantly suppressed tumor cell proliferation in the PDX mice model. In this study, we reported TYK2 directly regulated the phosphorylation of STAT3 in ESCC which was confirmed by TYK2 knockdown, overexpression, and kinase assays (Figs. 3c, d, and 4g, respectively). Taken together, these finding indicated that cirsiliol inhibited ESCC tumor growth in vivo by targeting TYK2. Previous studies have shown that STAT3 activation subsequently increased c-myc, Bcl-2, and Mcl-1 transcription, thereby inducing cancer cell proliferation and survival [34, 35]. Therefore, we assessed the p-STAT3 and c-myc, Bcl-2, and Mcl-1 protein levels in KYSE140 and KYSE450 cells after cirsiliol treatment. Data showed that the STAT3 phosphorylation was inhibited and the protein levels of its downstream pathway were decreased after cirsiliol treatment. In summary, cirsiliol inhibited the activity of TYK2, which in turn decreased the phosphorylation and dimerization of STAT3 which inhibited its effects on gene transcription (Fig. 7f).

## Conclusion

Our results demonstrated that TYK2 is a promising therapeutic target against ESCC. Cirsiliol could bind to TYK2 and inhibit its kinase activity. Cirsiliol also inhibited ESCC growth in vitro and in vivo by blocking TYK2/STAT3 signaling pathway. Our results suggest that the proper application of cirsiliol may be a beneficial chemo-preventive strategy for ESCC patients with high TYK2 levels.

## Supplementary Information

**Additional file 1: Supplementary 1.** (a) The TYK2 protein levels in each group of CDX mice tissues. (b) After knockdown TYK2, the cell cycle state in KYSE140 and KYSE450. (c) The variation of cell migration ability after TYK2 knockdown in KYSE140 and KYSE450. (d) The toxicity of cirsiliol on SHEE cell line. (e) The average body weight of mice in each group of LEG73 and LEG104 after treated by cirsiliol (*n* = 9 for LEG73, *n* = 8 for LEG104). (f) After continuous gavage administration for 2 weeks, the toxicity on mice body weight and white blood cell (WBC) were checked. Left panel: The average body weight of mice in each group after continuous treatment for 2 weeks for acute toxicity test (*n* = 3); right panel: the number of WBC after treated by cirsiliol. The mouse hematology was analyzed by PROKAN PE-6800. ANOVA was used for analysis in (b, c, d, e and f); no significant difference compared with control group was observed in (d, e and f).

## Data Availability

All data generated or analysed during this study are included in this published article [and its supplementary information files].

## Abbreviations

ESCC: Esophageal squamous cell carcinoma
PDX: Patient-derived xenograft
SPR: Surface plasmon resonance
STAT3: Signal transducer and activator of transcription 3
TPM: Transcription per million
TYK2: Tyrosine kinase 2
