# Supplementary figures and images for: Cirsiliol targets tyrosine kinase 2 to inhibit esophageal squamous cell carcinoma growth in vitro and in vivo
Source: J Exp Clin Cancer Res. 2021 Mar 17;40:105. doi: 10.1186/s13046-021-01903-z (PMC7972218; doi:10.1186/s13046-021-01903-z)

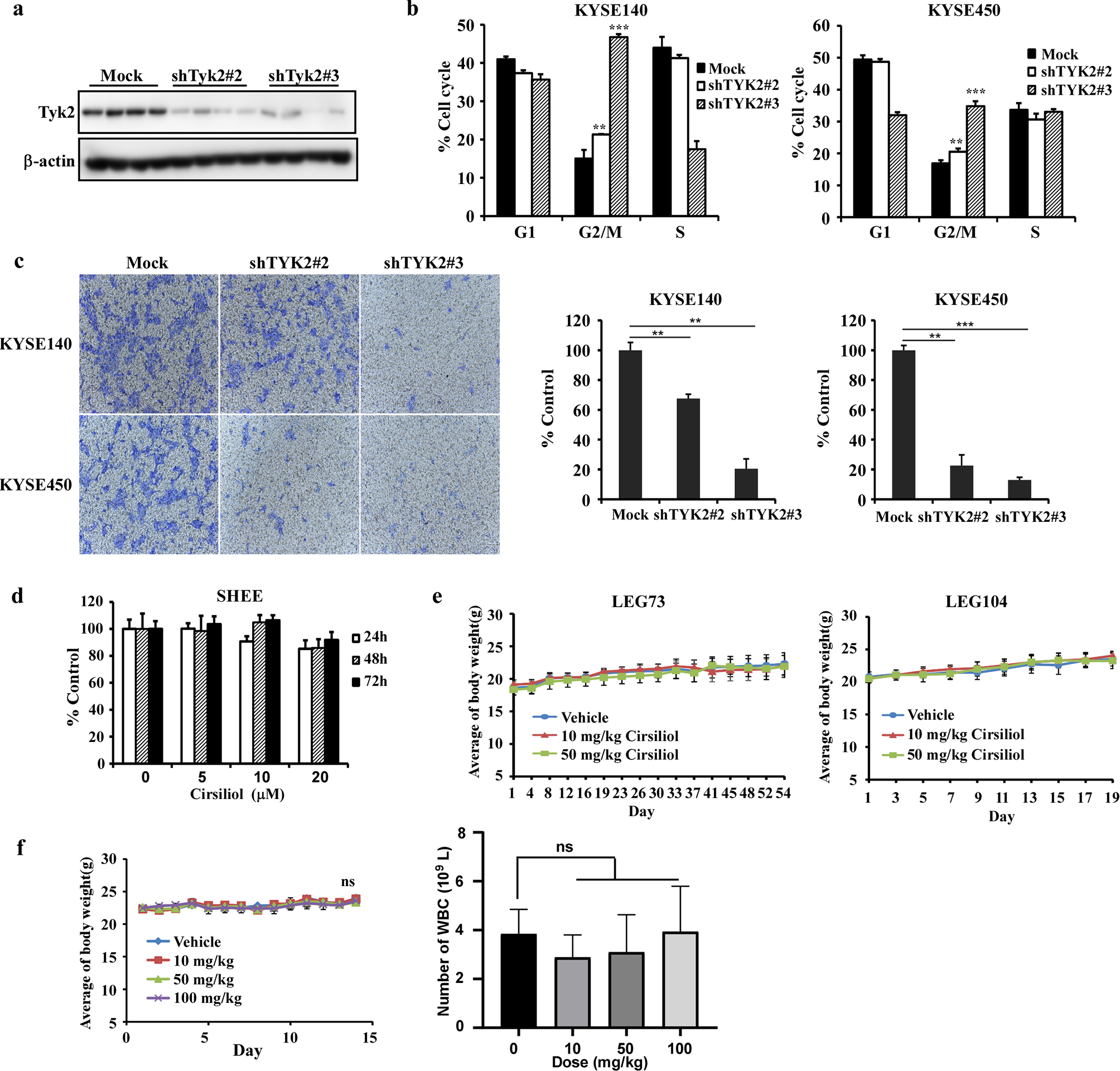

Supplement: Supplementary file 1 — Additional file 1: Supplementary 1. (a) The TYK2 protein levels in each group of CDX mice tissues. (b) After knockdown TYK2, the cell cycle state in KYSE140 and KYSE450. (c) The variation of cell migration ability after TYK2 knockdown in KYSE140 and KYSE450. (d) The toxicity of cirsiliol on SHEE cell line. (e) The average body weight of mice in each group of LEG73 and LEG104 after treated by cirsiliol (n = 9 for LEG73, n = 8 for LEG104). (f) After continuous gavage administration for 2 weeks, the toxicity on mice body weight and white blood cell (WBC) were checked. Left panel: The average body weight of mice in each group after continuous treatment for 2 weeks for acute toxicity test (n = 3); right panel: the number of WBC after treated by cirsiliol. The mouse hematology was analyzed by PROKAN PE-6800. ANOVA was used for analysis in (b, c, d, e and f); no significant difference compared with control group was observed in (d, e and f). [file 13046_2021_1903_MOESM1_ESM.tif]
